# Supplementary material for: Evaluating large language model performance and reliability in scoring picture description tasks for neuropsychological assessment
Source: PLOS Digit Health. 2026 Apr 21;5(4):e0001385. doi: 10.1371/journal.pdig.0001385 (PMC13098897; doi:10.1371/journal.pdig.0001385)
Supplement: S1 Appendix — (PDF) [file pdig.0001385.s001.pdf]

APPENDIX A

ID	age	gender	mmse	dx
**adrso005**	67	female	27	Control
**adrso008**	67	female	29	Control
**adrso012**	68	male	29	Control
**adrso021**	64	male	28	Control
**adrso027**	57	male	18	ProbableAD
**adrso031**	79	female	26	ProbableAD
**adrso032**	68	female	25	ProbableAD
**adrso049**	58	male	18	ProbableAD
**adrso063**	57	female	13	ProbableAD
**adrso070**	66	female	19	ProbableAD
**adrso093**	79	male	13	ProbableAD
**adrso156**	59	female	29	Control
**adrso161**	65	female	29	Control
**adrso164**	67	female	29	Control
**adrso180**	75	male	29	Control
**adrso183**	75	male	30	Control
**adrso216**	56	female	24	ProbableAD
**adrso218**	64	male	27	ProbableAD
**adrso220**	71	male	20	ProbableAD
**adrso229**	77	female	10	ProbableAD
**adrso237**	71	female	20	ProbableAD
**adrso260**	74	female	27	Control
**adrso286**	73	male	28	Control
**adrso309**	54	female	30	Control
**adrso312**	67	female	29	Control
