# Supplementary material for: Evaluating large language model performance and reliability in scoring picture description tasks for neuropsychological assessment
Source: PLOS Digit Health. 2026 Apr 21;5(4):e0001385. doi: 10.1371/journal.pdig.0001385 (PMC13098897; doi:10.1371/journal.pdig.0001385)
Supplement: S2 Appendix — (A) List of questions for Cookie Theft picture description task. (B) Few-Shot Example 1. (C) Few-Shot Example 2. (PDF) [file pdig.0001385.s002.pdf]

## S2 Appendix. Questions and examples provided to LLM and Human scorers.

### A. List of Questions

| # | Question |
|---|---|
| 1 | Did the participant describe a "boy"? |
| 2 | Did the participant describe a "cookie jar" or container of cookies? |
| 3 | Did the participant describe the action of someone reaching for or taking cookies? |
| 4 | Did the participant explicitly describe the location of the cookie jar (for example, that it is in a cabinet or in a high place)? |
| 5 | Did the participant describe a "stool" or ladder? |
| 6 | Did the participant describe the action of a boy or kid standing on a stool or ladder? |
| 7 | Did the participant explicitly describe the action of something (like a stool or ladder) tipping over? |
| 8 | Did the participant describe the action of someone (a boy or kid) about to fall off or falling off the stool, and/or that they are about to hurt themselves? |
| 9 | Did the participant describe a "girl"? |
| 10 | Did the participant describe the action of a girl reaching for, taking, or being given something? |
| 11 | Did the participant describe the girl holding a finger to her mouth, and/or acting like she is saying "be quiet" or is shushing? |
| 12 | Did the participant describe the girl smiling or laughing or having a positive emotion? |
| 13 | Did the participant describe a woman or mom? |
| 14 | Did the participant describe the action of someone washing or drying dishes? |
| 15 | Did the participant describe a sink (in general)? |
| 16 | Did the participant describe a sink that is overflowing? |
| 17 | Did the participant describe water overflowing onto the floor, or that there is water on the floor? |
| 18 | Did the participant describe the overall setting as being in a kitchen, using the word "kitchen"? |
| 19 | Did the participant describe a window? |
| 20 | Did the participant describe the curtains? |
| 21 | Did the participant describe the scene outside the window or outside, potentially including another house, a lawn, and bushes? |
| 22 | Did the participant describe dishes, cups, and/or plates on the counter or on the sink? |

**B. Few-Shot Example 1**

*Transcript*: "Let's see, a mom appears to be busy doing the dishes. Wow, she's having a problem. Uh, the sink is running over onto the floor. And meanwhile, while she's distracted, uh, the son and daughter are off into the cookies. He's grabbing a cookie, and she's taking one. And, oh no, he's about to fall. It's tipping over. Uh, umm. There's some grass and a house outside."

| # | Explanation | Answer |
|---|---|---|
| **1** | The participant mentioned 'the son,' which indicates a boy is described. | Yes |
| **2** | The participant mentioned cookies, but not specifically a cookie jar or container. | No |
| **3** | The participant said 'He's grabbing a cookie, and she's taking one,' which describes the action of reaching for or taking cookies out of the cookie jar. | Yes |
| **4** | The participant did not mention the location of the cookie jar (whether it is high up or in a cabinet). | No |
| **5** | The participant did not mention a stool or ladder. | No |
| **6** | Since a stool or ladder was not described, the boy (or kid) standing on one was not mentioned either. | No |
| **7** | The participant mentioned 'It's tipping over,' and while it only implies a stool or ladder is involved, the action of tipping over is described. | Yes |
| **8** | The participant said, 'Oh no, he's about to fall' which describes someone about to fall and potentially hurt themselves. | Yes |
| **9** | The participant mentioned 'the daughter,' which indicates a girl is described. | Yes |
| **10** | The participant stated 'He's grabbing a cookie, and she's taking one,' which describes the action of the girl reaching for or taking a cookie. | Yes |
| **11** | The participant did not describe the girl holding a finger to her mouth or acting like she is saying 'be quiet' or is shushing. | No |
| **12** | The participant did not describe the girl's emotions or mention her smiling, laughing, or having a positive emotion. | No |
| **13** | The participant mentioned 'a mom appears to be busy doing the dishes,' which indicates a woman or mom is described. | Yes |
| **14** | The transcript describes 'a mom appears to be busy doing the dishes', which indicates someone washing or drying dishes. | Yes |
| **15** | The participant mentioned 'the sink is running over,' which indicates a sink is described. | Yes |
| **16** | The participant stated 'the sink is running over onto the floor,' which indicates the sink is overflowing. | Yes |
| **17** | The participant mentioned 'the sink is running over onto the floor,' which indicates there is water on the floor. | Yes |
| **18** | The participant listed items that are typically in a kitchen (sink, dishes), but did not describe the overall setting explicitly as a kitchen. | No |
| **19** | The participant did not mention a window. | No |
| **20** | The participant did not describe curtains. | No |
| **21** | The participant mentioned 'there's some grass and a house outside,' which describes the scene outside. | Yes |
| **22** | The participant did not mention dishes, cups, and/or plates on the counter. | No |

### C. Few-Shot Example 2

*Transcript*: "There's a young boy, er, going in a cookie jar. Looks like it's in a cabinet. And there's a little girl, young girl. Uh, he's, he's in a cookie jar. And there's a stool that he is on. And it already is starting to fall over and so is the water in the sink. It's overflowing in the sink. I don't know about the, this thingy here, whether that's more than one I said. Uh, like the, the wife or, I mean, uh, the mother is near the girl and she's, uh, uh, She has, uh, uh, I can't think of the, she has the, she's trying to wipe, wipe dishes. And stop the water from going out."

| # | Explanation | Answer |
|---|---|---|
| 1 | The participant explicitly mentions a 'young boy' going into a cookie jar, indicating the presence of a boy. | Yes |
| 2 | The participant mentions the boy going 'in a cookie jar,' which indicates the presence of a cookie jar. | Yes |
| 3 | The description of the boy 'going in a cookie jar' suggests the action of reaching for or taking cookies. | Yes |
| 4 | The location of the cookie jar is noted as being 'in a cabinet'. | Yes |
| 5 | The participant mentions 'there's a stool that he is on,' indicating the presence of a stool. | Yes |
| 6 | The participant directly states that the boy is 'on' a stool. | Yes |
| 7 | The participant mentions 'it already is starting to fall over.' Because in the previous sentence he mentioned 'a stool', we know the 'it' is referring to the stool 'starting to fall over'. | Yes |
| 8 | Although the stool is mentioned as starting to fall over, there is no explicit mention of the boy about to fall off or that he is about to hurt himself. | No |
| 9 | The participant mentions 'there's a little girl, young girl,' indicating the presence of a girl. | Yes |
| 10 | There is no mention of the girl reaching for a cookie or taking a cookie. | No |
| 11 | There is no mention of the girl holding a finger to her mouth or acting like she is saying 'be quiet' or shushing. | No |
| 12 | There is no mention of the girl smiling, laughing, or exhibiting a positive emotion. | No |
| 13 | The participant mentions 'the wife or, I mean, uh, the mother,' indicating the presence of a woman or mom. | Yes |
| 14 | The participant mentions the mother is 'trying to wipe, wipe dishes,' indicating the action of someone washing or drying dishes. | Yes |
| 15 | The participant explicitly mentions 'the sink' when talking about water overflowing, indicating the presence of a sink. | Yes |
| 16 | The participant explicitly states 'the water in the sink. It's overflowing in the sink,' indicating an overflowing sink. | Yes |
| 17 | While the sink is mentioned as overflowing, there is no explicit mention of water overflowing onto the floor or that there is water on the floor. | No |
| 18 | The participant did not describe the overall setting explicitly as a kitchen. | No |
| 19 | The participant does not mention a window in the description. | No |
| 20 | The participant does not describe curtains. | No |
| 21 | There is no description of the scene outside the window or outside in general. | No |
| 22 | While the participant mentions the mother wiping dishes, there is no explicit mention of dishes, cups, and/or plates on the counter. | No |
