# Supplementary material for: Evaluating large language model performance and reliability in scoring picture description tasks for neuropsychological assessment
Source: PLOS Digit Health. 2026 Apr 21;5(4):e0001385. doi: 10.1371/journal.pdig.0001385 (PMC13098897; doi:10.1371/journal.pdig.0001385)
Supplement: Attachment — Submitted filename: Response_to_Reviewers.pdf [file pdig.0001385.s004.pdf]

RESPONSE TO REVIEWERS

Many thanks to all the reviewers for their time and expertise in reviewing this manuscript. This manuscript was recognized for its innovative and timely findings with strong clinical relevance. The issues raised by the reviewers have each been explored and addressed, and detailed here:

1. *The analysis was based on a subset of 25 participants selected from the DementiaBank corpus. Author stated that in line76-77: "The reduced size of the subset was necessary due to the requirement that each recording be manually transcribed, processed, and scored." Please provide the explanation of the "requirement" with reference. Why only 25 with 13 cognitively normal and 12 cognitively impaired? How those 25 were selected from the 237 total sample?*

AUTHOR RESPONSE: The subset size of 25 participants was determined by practical resource constraints related to the labor-intensive nature of 1) manual verification and correction of automated transcripts (roughly 15-20 minutes per transcript), 2) independent scoring by three human raters (approximately 20-30 minutes per transcript per rater), and 3) consensus meeting discussions (approximately 15-20 minutes per participant). This resulted in approximately 50 hours of human labor for the current sample size. The 25-participant sample was selected to balance adequate statistical power for within-subjects comparisons across models and prompt strategies, roughly equal representation of cognitively normal (n=13) and impaired (n=12) participants, and practical feasibility given resource constraints.

Participants were randomly selected from the DementiaBank corpus with stratification by cognitive status and matching where possible on available demographic variables (age and sex). I have added this explanation to the manuscript along with a power analysis (see response to comment 2).

> *Lines 71-79*: The data used in this study was obtained from the DementiaBank [13] ADReSSo challenge [14], which consists of speech recordings of picture description tasks using the Cookie Theft image from the Boston Diagnostic Aphasia Examination [1]. Recordings were collected from both cognitively normal participants as well as cognitively impaired patients with a diagnosis of Alzheimer's disease. A subset of 25 participants, 13 classified as cognitively normal and 12 as cognitively impaired, were selected from the sample of 237 total participants with collected audio recordings of picture description tasks, stratified by cognitive status and matched where possible on age and gender. This sample size was determined by practical resources constraints: each recording required manual transcript verification, independent scoring by each of three human raters, and consensus discussion with a fourth neutral rater, totalling approximately 50 hours of human labor. This sample size provided adequate statistical power for within-subjects comparisons across the five LLMs and four prompt strategies (see 2.5.4 Statistical Power). Participant characteristics were controlled between groups where possible, given the limited demographics available; only age, sex, MMSE score, and a probable diagnosis were present in the dataset. The IDs and other characteristics of the selected participants can be found in **Appendix A**.

2. *The interrater reliability among human raters (ICC = 0.347) was notably low, which raises concerns about the validity of the scoring reference. Some justification or sensitivity analysis may be needed. Although the MAE and ICC are reported, author may consider reporting the effect sizes (e.g., Cohen's d) alongside p-values in all inferential statistics with such small sample size. Also, a power analysis to support the adequacy of the sample size is needed.*

AUTHOR RESPONSE: The low human ICC is consistent with existing literature on subjective scoring of verbal tasks (Purpura et al., 2015) and is one of the key motivators for automating the scoring procedure. It is also the reason why when determining “ground truth” via the consensus process, a fourth independent rater was included. Nonetheless, as mentioned in the Limitations section, there were no significant differences found between raters despite the low ICC score. Additional language supporting the layperson as a rater was added:

> *Lines 189-191:* The layperson with additional training was chosen to increase real-world validity by approximating the performance of a newly trained neuropsychologist or one who does not frequently score these tasks.

I agree that including the effect sizes would improve the paper. For each significant comparison, Cohen’s *d* or Eta-squared has been included. Power analysis supporting the sample size has also been included in section *2.5.4*:

> *Lines 225*-231: Power analyses were conducted using G*Power 3.1.9.7 for the primary analysis comparing LLM and human rater performance. For repeated measures ANOVAs comparing prompt strategies (4 levels) and model types (5 levels) with α = 0.05 and n= 25, and assuming moderate correlations among repeated measures (r = 0.5), the study achieved 80% power to detect effect sizes of *d* = 0.3-0.4 (small-to-medium effects). For human vs LLM comparisons using two-level repeated measures ANOVAs, 80% power was achieved for *d*=0.6, (medium effect).

3. *There is minimal discussion of participant demographics beyond age, gender, and MMSE scores. For example, the language variation and education can influence performance on picture description tasks and LLM interpretability. A brief discussion of these covariates and their potential impact on the results are needed.*

AUTHOR RESPONSE: This is an unfortunate limitation of the *DementiaBank* corpus. Only age, sex, MMSE, and probable diagnosis was provided. This has been clarified in the Methods section 2.1, line 84, as well as the Limitation section:

> *Lines 471-476:* An important limitation is the lack of comprehensive demographic data in the DementiaBank corpus. Education level, linguistic background, and socioeconomic factors were not available, limiting our ability to assess how these variables might influence LLM scoring accuracy. Prior research has demonstrated that dialectical variation can affect picture description scoring systems [8], and educational attainment influences linguistic complexity in cognitive assessments. Future studies should examine LLM performance across diverse demographic groups.

4. *Line 89:” Each content unit was also formed into a simple True/False question and used by both the human raters as well as the LLM models to provide scores for the presence or absence of each content unit.” Please include a table for the list of the content unit questions used in the data coding process. In addition, the item-level error analysis, such as which content units caused most discrepancy could be included in the data analysis.*

AUTHOR RESPONSE: While both of these are currently addressed in the current manuscript (content units are found in Appendix B and item-level error analysis in Table 3), it is clear that the signposting is not adequate. I have added language clarifying the locations of these.

5. *Although the paper discusses cost differences between models (e.g., Claude Opus vs GPT-4o), it lacks quantitative detail. Reporting actual processing time, token counts, and estimated dollar costs per transcript would provide clearer guidance for real-world deployment.*

AUTHOR RESPONSE: I agree that this would be interesting to compare, thank you for the suggestion. I have calculated average token amounts and added language comparing estimated dollar costs per transcript to section 4.1. However, processing time data was not collected when originally processed and so would require all models to be re-run, requiring considerable monetary and time costs.

> *Lines 366-369*: To enable cost comparison, we calculated the average transcript length used in this study as 147.5 token, with the system prompt for few-shot prompting at 2934 tokens (3081.5 input tokens), with the average output including explanations and scoring producing 504.4 output tokens. Using these averages, Opus costs approximately $0.0841 per transcript, in comparison to GPT4o's $0.0127 per transcript.

6. *Consider clarifying the low ICC for GPT-4o reported in Table 2 (0.110). This appears inconsistent with other reported findings and may need to be checked or contextualized.*

AUTHOR RESPONSE: I believe this may be a misreading of Table 2. No ICC of 0.110 was reported; instead, the human raters were assigned a p-value of 0.110.

7. *Including a table with examples of scoring errors (model vs. human) would help illustrate the types of disagreements observed.*

AUTHOR RESPONSE: Described in section 3.5, only three questions were significantly different between human and machine raters. While other discrepancies were present between individual human raters and model scoring, no significant differences were found. For these three questions, a handful of examples of errors were chosen to best illustrate differences:

> *Lines 304-319:* Question 7, involving the stool tipping over, was occasionally scored as present in error when only the action of the boy falling off the stool is described. For example, subject 229's statement *"I thought the kid's going to fall off the stool"* was commonly misinterpreted by both human raters and most models as describing the stool about the fall, instead of the boy. The opposite was observed for question 8, which describes the action of the boy falling off the stool; while occurring less often than question 7, both human raters and models occasionally correctly marked present the boy standing on the stool (question 6) and the stool being described as tipping over (question 7), followed by scoring question 8 as true even when the action of falling off was not described. In subject 286's transcript, they describe *"this boy's on the stool getting cookies. His stool's about to fall"* which does not describe the action of the boy himself falling. Despite this, it was incorrectly rated as present by 1/3 human raters and GPT-4o. Finally, question 16 ("did the participant describe a sink that is overflowing?") was often scored as present when only water was described as being on the floor (question 17) rather than the sink being the thing that is overflowing; for example, subject 218 describes *"the water's spilling on the floor"* which was scored incorrectly by 1/3 human raters and at least one run of every model.

8. *The discussion could benefit from a brief reflection on the ethical implications of using LLMs in cognitive assessment (e.g., fairness, bias, transparency).*

AUTHOR RESPONSE: I agree that this would be an important addition to the manuscript. I have added a new subsection 4.5. *Ethical considerations* to the Discussion that outlines cultural fairness, bias, and transparency.

9. *Reference formatting is inconsistent in a few places; ensure alignment with journal style guidelines. Table formatting and figure references (e.g. Figure 1) should align with journal style guidelines as well.*

AUTHOR RESPONSE: Formatting has been corrected and should now properly align with style guidelines.
