# Supplementary material for: Evaluating large language model performance and reliability in scoring picture description tasks for neuropsychological assessment
Source: PLOS Digit Health. 2026 Apr 21;5(4):e0001385. doi: 10.1371/journal.pdig.0001385 (PMC13098897; doi:10.1371/journal.pdig.0001385)
Supplement: Attachment — Submitted filename: PDIG-D-25-00277-review report.docx [file pdig.0001385.s003.docx]

**Manuscript Title:**
*Evaluating Large Language Model Performance and Reliability in Scoring Picture Description Tasks for Neuropsychological Assessment*

**General Comments**
This manuscript investigates the feasibility and reliability of using large language models (LLMs) to automate scoring of the Cookie Theft picture description task, a widely used neuropsychological tool. While the manuscript makes a strong and original contribution, several methodological and demographic reporting issues should be addressed before publication. In particular, more robust justification of the scoring reference standard, detailed statistical reporting, and expanded cost/efficiency analysis are needed to enhance the study’s impact and reproducibility.

**Specific comments**
1. The analysis was based on a subset of 25 participants selected from the DementiaBank corpus. Author stated that in line76-77: “The reduced size of the subset was necessary due to the requirement that each recording be manually transcribed, processed, and scored.” Please provide the explanation of the “requirement” with reference. Why only 25 with 13 cognitively normal and 12 cognitively impaired? How those 25 were selected from the 237 total sample?

2. The interrater reliability among human raters (ICC = 0.347) was notably low, which raises concerns about the validity of the scoring reference. Some justification or sensitivity analysis may be needed. Although the MAE and ICC are reported, author may consider reporting the effect sizes (e.g., Cohen’s d) alongside p-values in all inferential statistics with such small sample size. Also, a power analysis to support the adequacy of the sample size is needed.

3. There is minimal discussion of participant demographics beyond age, gender, and MMSE scores. For example, the language variation and education can influence performance on picture description tasks and LLM interpretability. A brief discussion of these covariates and their potential impact on the results are needed.

4. Line 89:” Each content unit was also formed into a simple True/False question and used by both the human raters as well as the LLM models to provide scores for the presence or absence of each content unit.” Please include a table for the list of the content unit questions used in the data coding process. In addition, the item-level error analysis, such as which content units caused most discrepancy could be included in the data analysis.

5. Although the paper discusses cost differences between models (e.g., Claude Opus vs GPT-4o), it lacks quantitative detail. Reporting actual processing time, token counts, and estimated dollar costs per transcript would provide clearer guidance for real-world deployment.

6. Consider clarifying the low ICC for GPT-4o reported in Table 2 (0.110). This appears inconsistent with other reported findings and may need to be checked or contextualized.

7. Including a table with examples of scoring errors (model vs. human) would help illustrate the types of disagreements observed.

8. The discussion could benefit from a brief reflection on the ethical implications of using LLMs in cognitive assessment (e.g., fairness, bias, transparency).

9. Reference formatting is inconsistent in a few places; ensure alignment with journal style guidelines. Table formatting and figure references (e.g. Figure 1) should align with journal style guidelines as well.
